# Comparative effectiveness of treatment with the first TNF antagonist in monotherapy, the first TNF antagonist plus one conventional synthetic disease-modifying antirheumatic drug, and the first TNF antagonist plus two or more conventional synthetic disease-modifying antirheumatic drugs in patients with rheumatoid arthritis

**DOI:** 10.1186/s13075-016-1137-4

**Published:** 2016-11-08

**Authors:** Blanca Hernández-Cruz, Esther Márquez-Saavedra, Rafael Caliz-Caliz, Federico Navarro-Sarabia

**Affiliations:** 1 Rheumatologist and Investigator, Rheumatology Clinical Unit, Virgen de la Macarena University Hospital, Andalusian Health Service, Seville, Spain; 2 Pharmaceutical Supplies and Services, Central Services, Reina Sofia University Hospital, Andalusian Health Service, Cordoba, Spain; 3 Rheumatologist, Head of Service., Virgen de las Nieves University Hospital, Andalusian Health Service, Granada, Spain; 4 Servicio de Reumatología. Planta Semisótano, Hospital Universitario Virgen Macarena, Av, Dr Fedriani No 3, Seville, CP 41007 Spain

**Keywords:** RA, csDMARDs, Glucocorticoids, TNF antagonist

## Abstract

**Background:**

Rheumatoid arthritis (RA) patients are treated with a mean of 3–4 conventional synthetic disease-modifying antirheumatic drugs (csDMARDs) with or without glucocorticoids (GCs), before the first biologic prescription. The main reasons for change are inefficacy in 30–40 % of patients, and toxicity ≈ 10 %. Thus, they are treated with the first TNF antagonists in monotherapy. The aim of this study was to analyse the csDMARD and GC prescription patterns before and during treatment with the first TNF antagonist, and compare their effectiveness in three groups of patients.

**Methods:**

An observational, prospective, multicentre study in common clinical practice was designed. Treating rheumatologists recorded patient variables, including previous and concomitant csDMARDs and GCs in a database. The data were analysed using descriptive, inferential and multivariate statistics.

**Results:**

There were 1136 patients included; 21 % received the first TNF antagonist in monotherapy, 67 % received the first TNF antagonist plus one csDMARD, and 12 % the first TNF antagonist plus two or more csDMARDs. Most patients were female (73 %), RF+, and ACPA+, and had erosions; mean age was 53.2 (±13.0) years, and duration of disease was 9.1 (±7.6) years. They had high activity with DAS28 of 5.8 ± 1.1, and poor physical function with HAQ of 1.43 ± 0.63, and significant differences between groups in clinical variables and comorbidities; 94 % had received treatment with GCs, MTX, LFN, or SSZ at any time before the first TNF antagonist, 5 % (*n* = 52) had been treated with CLQ or HCLQ, and 1 % (*n* = 13) had received neither GCs nor csDMARDs. Before the first TNF antagonist, the drugs most commonly used were GCs (78 %), MTX (50 %), LFN (44 %), and SSZ (21 %). Concomitantly with the first TNF antagonist, 977 patients (85 %) were receiving GCs, MTX, LFN, or SSZ; 15 % (*n* = 173) received their first TNF antagonist without any concomitant GCs or csDMARDs, true monotherapy, and 6 % received their first TNF antagonist with GCs. The drug most commonly used at the time of first TNF antagonist initiation was MTX (58 %). All treatment groups had clinically and statistically significant improvements in DAS and HAQ scores. Effectiveness analysis (controlling for confounders) showed mean drug survival of 16.7, 20.1 and 11.7 months in each group, respectively (*p* < 0.001). The model that best explained a good EULAR response included the baseline and 6-month DAS28.

**Conclusions:**

The three groups of patiernts, have different comorbidities and disease characteristics. Treatment with low or very low doses of GCs is common. True monotherapy with the first TNF antagonist without prednisone or csDMARDs is infrequent. After controlling for potential confounders, effectiveness was a little different.

## Background

Conventional synthetic disease-modifying antirheumatic drugs (csDMARDs) with or without glucocorticoids (GCs) are the first line of the treatment for rheumatoid arthritis (RA) in the T2T strategy and in most of the recommendations for RA treatment [1, 2], and see https://www.nice.org.uk/guidance/cg79/chapter/Recommendations#pharmacological-management [3]. They can be used as sequential monotherapy or in combination, and in all of them, methotrexate (MTX) is the “anchor drug” [4].

Before the first biologic drug is prescribed, the patients are treated with a mean of 3–4 csDMARDs, mainly MTX, leflunomide (LFN), sulfasalazine (SSZ) and antimalarial drugs [5]. The main reasons for changing this therapy are inefficacy in 30–40 % of patients with early RA [6], and toxicity in about 10 % of patients [4–7]. Thus, between 10 % and 30 % of patients seen in clinical practice are on inefficacious treatment or have serious comorbidities or toxicity that rule out the use of MTX, LFN, and SSZ [8]. Some of these comorbidities are hepatitis B or C virus infections, abnormal liver function, severe anaemia, leukopenia or thrombocytopenia, severe or recurrent infections, past history of cancer, or multiple sclerosis. Thus, up to a third of patients are treated with biologic disease-modifying antirheumatic drugs (bDMARDs) in monotherapy (i.e., without any concomitant csDMARD) [8–14].

Summaries of the product characteristics of bDMARDs, with the exception of tocilizumab (TCZ), and clinical practice guidelines, clinical trials meta-analyses, and clinical practice registries recognise that a TNF antagonist plus csDMARD combination therapies are associated with better outcomes and with greater retention rates. [1, 8–14]. In controlled clinical trials (CCT), bDMARD monotherapy (except for tocilizumab (mainly anti-TNF)) had lower efficacy and survival rates when compared with combination therapies [13, 14]. European League Against Rheumtism (EULAR) recommendations are clear on this point, and emphasize the complexity of patient management in RA and the benefits of GCs at low doses [1, 2, 15]. GCs at low or very low doses are commonly used in clinical practice in combination with csDMARDs, particularly in patients with early disease, and there is a growing body of evidence supporting their role as cost-effective csDMARDs [16, 17].

As TNF antagonist are frequently used in monotherapy for reasons of safety in patients with adult-onset RA treated within the Andalusian Health Service, a registry was designed with the following objectives:

1. To understand and compare clinical features of patients who receive the first TNF antagonist monotherapy, against the patients who receive the first TNF antagonist plus one csDMARD, against the patients who receive the first TNF antagonist plus two or more csDMARDs.
2. To determine the prescription patterns of csDMARDs with high efficacy (MTX, LFN, and SSZ) and GCs before the first TNF antagonist prescription.
3. To identify csDMARD and GC prescription patterns at initiation of the first TNF antagonist.
4. To assess the effectiveness of the first TNF antagonist in the three groups of patients, using EULAR response, and improvement in health assessment questionnaire (HAQ) scores, survival curves and multiple logistic regression analysis.

## Methods

### Design

This was an observational, prospective, multicentre, analytical study.

### Inclusion criteria

Inclusion criteria were patients over 18 years of age, able to complete follow-up questionnaires, fulfilling criteria for RA published in 1987 [18], and treated with at least one dose of any TNF antagonist (adalimumab, infliximab, etanercept) approved for use in patients with moderately active RA in accordance with the Second Consensus Document on the Use of Biologic Agents in RA of the Spanish Rheumatology Society [19]. This consensus document states that a patient should have failed treatment with two or more csDMARDs for at least 3 months, or have developed toxicity to the administered drugs in order to initiate therapy with a TNF antagonist.

Patients with diagnoses other than RA, duplicated registration, or those whose data were insufficient to calculate clinically relevant outcomes were excluded from the study. The study was approved by the Ethical and Human Research Committee of the Virgen de la Macarena Hospital Area, and conducted in accordance with The Declaration of Helsinki [20] and the Good Clinical Practice Guidelines (http://www.ich.org/products/guidelines/efficacy/efficacy-single/article/good-clinical-practice.html). The SAS, the public healthcare system in the Autonomous Community of Andalusia, prescribes and reimburses approximately 80 % of bDMARDS, while the remaining 20 % are prescribed by private medical clinics. Registry completion was mandatory to obtain the bDMARD from the SAS. All rheumatology departments of Andalusia were invited to take part in the registry. Participating rheumatologists received training on the study objectives and on the different operative definitions used in this project. Inefficacy was defined according to the judgement of the treating rheumatologist. Serious adverse events were defined as death, hospitalization, or life-threatening for the patient. Non-serious adverse events were defined as non-serious clinical conditions leading to discontinuation of therapy with DMARDs.

A treating rheumatologist and/or a research fellow at each centre had access to a specifically designed website, and prospectively recorded the following information: sociodemographic and RA-related variables, with particular emphasis on the patient’s disease activity, physical function, previous and concomitant therapy with csDMARDs and GCs, current therapy with csDMARDs and GCs at initiation of therapy with the biologic agent, reasons for discontinuation of csDMARDs or GCs or switch, and comorbidities. The relevant comorbidities were: secondary Sjögren’s syndrome, interstitial lung disease, rheumatoid vasculitis, amyloidosis, latent or active tuberculosis, past history of tuberculosis, purified protein derivative skin reaction (PPD) test, hepatitis B or C infection, HIV infection, other severe or recurrent infections, previous cancer, history of heart failure, and demyelinating disease. The number of comorbidities and mortality were also registered.

Patients were divided into three groups according to the type of treatment they were receiving: first TNF antagonist monotherapy; first TNF antagonist plus one csDMARD; and a first TNF antagonist plus two or more csDMARDs. From the website the data were recorded in an Excel spreadsheet. The Excel spreadsheet was cleaned and the data were verified with the clinical charts, for subsequent processing using STATA v 10.0 software.

Central tendency and dispersion measures were calculated in the first phase of the analysis and graphic analysis was performed. The three groups were subsequently compared using analysis of variance (ANOVA) for quantitative variables with a Gaussian distribution and/or the Kruskal − Wallis test for quantitative variables with a non-Gaussian distribution or ordinal variables. In the case of significant differences, the Mann–Whitney *U* test or Student’s *t* test with Bonferroni correction for multiple comparisons was used to analyse specific differences between two of the three groups. For nominal variables, contingency tables were produced and the chi-squared or fisher exact test was applied. In all cases the most pragmatic statistical analysis was performed.

For effectiveness analyses the primary outcome was good/moderate EULAR response with a first anti-TNF therapy, which was defined as the length of time the patients continued to receive their first anti-TNF therapy; patients were censored at the treatment stop date, date of death or date of the last follow up, whichever came first. Kaplan–Meier survival curves were used to describe persistence with anti-TNF therapy. Also logistic regression was used to identify predictive models of EULAR good response. Survival analysis and logistic regression were performed in a crude way, and adjusted by age, sex, disease duration, rheumatoid factor (RF) positivity, anti-citrullinated protein antibodies (ACPA) positivity, baseline disease activity score in 28 joints (DAS28), baseline health assessment questionnaire (HAQ), erosions, and number of comorbidities. In a third phase a propensity score was created using the same variables in order to adjust for severity of the disease.

Missing values were imputed using three techniques: (1) using the mean value of the previous observations, (2) using the value of the last observation, and (3) looking at the pattern of missing values in the time series, and calculating these values.

## Results

The registry was active from 15 May 2008 to 31 March 31 2012. During this period, 20 physicians from 18 rheumatology departments, and 2 internal medicine departments recorded data from 1237 patients treated with their first biologic agent. There were 101 patients (8 %) excluded from the registry for various reasons: 47 patients did not initiate treatment, 28 had duplicate registrations, 14 patients started rituximab, 6 patients had received abatacept as their first biologic agent, and 6 were excluded due to lack of indication. Results of 1136 patients with adult-onset RA are presented. Of these patients, 21 % received their first TNF antagonist in monotherapy, 67 % received their first TNF antagonist plus one csDMARD, and 12 % were treated with a first TNF antagonist plus two or more csDMARDs (Table 1).

**Table 1:** Rheumatoid arthritis characteristics at baseline

	First TNF antagonist monotherapy		First TNF antagonist plus one csDMARD		First TNF antagonist plus two or more csDMARDs		Total		P*
n, %	234	21	766	67	136	12	1.136	100	
Gender, female n, %	175	75	557	73	99	73	831	73	0.8
Rheumatoid factor (RF)+ n, %	183	78	619	81	104	77	906	80	0.4
ACPA + n, % *	47/71	66	330/431	77	47/70	67	424/ 572	74	0.06
RF- and ACPA- n, %	12	5	64	8	14	10	90	8	0.07
Erosions n, %	195	83	622	81	100	74	917	81	0.05
Rheumatoid nodules n, %	50	21	189	25	18	13	257	23	0.01
Joint arthroplasty n, %	15	6	66	9	3	2	84	7	0.02
Number of comorbidities n, %									
0	67	30	159	22	51	42	277	26	0.003
1	32	14	72	10	12	10	116	11	
≥2	124	56	481	68	57	48	662	63	
Anti-hepatitis B antibody+ n, %	2/47	4	7/194	4	0/26	0	9/267	3	0.2
Anti-hepatitis C antibody+ n, %	6/46	13	6/195	3	2/26	8	14/267	5	0.05
PPD skin reaction + (≥5 mm) n, %	24	10	120	16	17	13	161/1.112	14	0.09
Secondary Sjögren’s syndrome n, %	44	19	128	17	17	12	189	17	0.2
Interstitial lung disease n, %	16	7	25	3	1	0.7	42	4	0.007
Recurrent infections n, %	5	2	11	1	1	0.7	17	2	0.54
Past cancer n, %	4	2	5	0.6	0	0	9	0.8	0.1
Mean ± SD									
Age (years) mean ± SD	55.9	±13.7	52.8	±13.4	50 .7	±11.1	53.2	±13.3	0.0001
Duration of disease (years) mean ± SD	9.4	±8.0	9.0	±7.5	8.8	±7.3	9.1	±7.6	0.8
Number of comorbidities mean ± SD	2.2	±2.3	2.7	±2.4	1.9	±2.3	2.5	±2.4	0.0001
Tender joint count (0–28) mean ± SD	10.9	±6.4	10.3	±6.6	11.6	±6.9	10.6	±6.6	0.0003
Swollen joint count (0–28) mean ± SD	9.0	±6.1	7.9	±5.5	8.5	±5.8	8.2	±5.7	0.02
Pain visual analogue scale (0–100) mean ± SD	61.9	±19.3	63.5	±22.2	66.0	±20.8	63.4	±21.5	0.01
DAS28-ESR mean ± SD	5.9	±1.0	5.7	±1.1	5.7	±1.0	5.8	±1.1	0.03
HAQ score (0–3) mean ± SD	1.40	±0.58	1.44	±0.65	1.6	±0.59	1.46	±0.63	0.0003
ESR (mm) mean ± SD	45.7	±24.7	42.1	±25.8	32.7	±21.5	41.8	±25.3	0.0001
C-reactive protein (mg/L) mean ± SD	19.3	±21.4	15.7	±21.5	15.8	±19.4	16.5	±21.2	0.0001

*csDMARD* conventional synthetic disease-modifying antirheumatic drug, *ACPA* anti-citrullianted protein antibodies, *PPD* purified protein derivative skin reaction, *DAS28-ESR* disease activity score in 28 joints-erythrocyte sedimentation rate, *HAQ* health assessment questionnaire.

*This variable was not assessed in all patients

Differences between groups were observed in patient characteristics, percentages of patients with joint arthroplasty, number of comorbidities, positive hepatitis virus antibodies, and positive tuberculin test results (Table 1). In addition to the comorbidities shown in the table, there were 13 patients (1 %) with rheumatoid vasculitis, of whom 3 (1 %) were patients in the group treated with the first TNF antagonist in monotherapy, 9 (1 %) were in the group treated with the first TNF antagonist plus one csDMARD, and 1 (0.7 %) was in the group treated with the first TNF antagonist plus two or more csDMARDs (*p* = 0.8); in the same groups respectively, there were 2 patients (0.8 %), 4 patients (0.5 %), and 0 patients (0 %) with amyloidosis, respectively *p* = 0.5), and 1 patient (0.4 %), 4 patients (0.5 %), 0 patients (0 %) with previous heart failure (*p* = 0.6). Previous tuberculosis was identified in 6 patients (3 %), 24 patients (3 %) and 0 patients (0 %), respectively (*p* = 0.08). In the group treated with the first TNF antagonist plus one csDMARD there was one patient with HIV infection and one with demyelinating disease. Neither of these patients died.

**Fig. 1:**
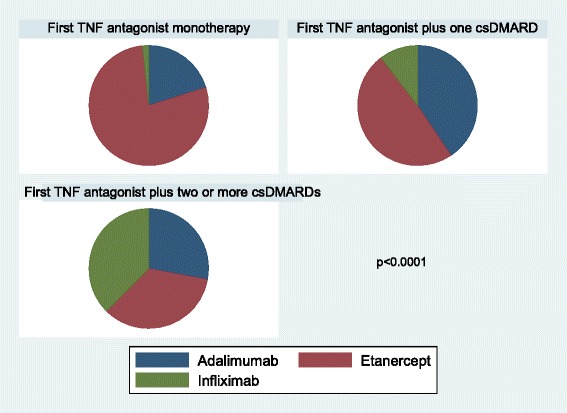
First prescribed biologic disease-modifying antirheumatic drug (bDMARD). *csDMARD* conventional synthetic DMARD

Types of first TNF antagonist are shown on Fig. 1. As expected, and in accordance with the summaries of product characteristics and treatment recommendations, most patients received etanercept as their first TNF antagonist monotherapy, and significant differences were observed in the type of TNF antagonist used within each treatment group, *p* < 0.0001.

Among all patients, 94 % had received treatment with GCs, MTX, LFN, or SSZ at any time before the TNF antagonist prescription (Table 2); 6 % had not received these drugs, 5 % (*n* = 52) had been treated with antimalarial agents, and only 1 % (*n* = 13) had received neither GCs nor csDMARDs. Most patients had received combination therapies (*n* = 761; 66 %). The drugs most commonly used in monotherapy or in combination were GCs, either prednisone (PDN) or equivalent, used in 78 % of patients, followed by MTX (50 %), LFN (44 %), and SSZ (21 %). The most frequent combination was GC plus MTX, LFN, or SSZ, used in 31 % (*n* = 357) of patients, followed by a GC plus two csDMARD 23 % (*n* = 267). Combinations of csDMARDs without GCs were less frequent: 4 % of patients were treated with MTX + SSZ + LFN + PDN. Inefficacy of the drug was the primary reason for MTX, LFN, and SSZ discontinuation in over half of the patients. Non-serious adverse events were the second reason for discontinuation of these csDMARDs in 40 %, 36 %, and 40 % of patients, respectively. The frequency of serious adverse events was very low.

**Table 2:** Prescription patterns of csDMARDs (MTX, LFN y SSZ) and GCs before the firs TNF antagonist

Treatment	First TNF antagonist monotherapy		First TNF antagonist plus one csDMARD		First TNF antagonist plus two or more csDMARDs		Total		P*
	*n*	%	*n*	%	*n*	%	*n*	%	
	234	21	766	67	136	12	1.136	100	
Previous use of DMARDs (MTX, LFN, SSZ) and/or PDN) (n , %)	229	98	712	93	130	96	1,071	94	
None (n, %)	5	2	54	7	6	4	65	6	0.0001
One (n, %)									< 0.001
PDN	8	3	139	18	76	56	223	20	
MTX + PDN	47	20	122	16	9	7	178	16	
LFN + PDN	17	7	106	14	8	6	131	11	
LFN	1	0.4	41	5	2	1	44	4	
SSZ + PDN	3	1	29	4	12	9	44	4	
MTX	5	2	33	4	1	0.7	39	3	
SSZ	4	2	11	1	1	0.7	16	1	
Total (MTX, LFN, SSZ)	77	33	342	45	33	24	452	40	
Total PDN	75	32	396	52	105	77	576	51	
Two (n, %)									< 0.001
LFN + MTX + PDN	67	29	89	12	12	9	168	15	
LFN + MTX	16	7	26	3	2	1	44	4	
SSZ + MTX + PDN	15	6	31	4	3	2	49	4	
SSZ + LFN + PDN	4	2	38	5	2	1	44	4	
SSZ + MTX	8	3	14	2	1	0.7	23	2	
SSZ + LFN	1	0.4	6	0.7	0	0	7	0.6	
Total (MTX, LFN, SSZ)	111	48	204	27	20	15	335	30	
Total PDN	86	37	158	21	17	12	218	20	
Three (n, %)									< 0.001
MTX + SSZ + LFN	6	2	6	0.7	1	0.7	13	1	
MTX + SSZ + LFN + PDN	27	12	21	3	0	0	48	4	
Total (MTX, LFN, SSZ)	33	14	27	3	1	0.7	61	5	
Total PDN	27	12	21	3	0	3	48	4	
Previous exposure to MTX (n, %)	191	83	343	45	29	21	563	50	0.03
Reason for discontinuation									
Inefficacy	92	48	212	62	16	55	320	57	
Non-serious adverse event	91	48	121	35	13	45	225	40	
Serious adverse event	8	4	10	3	0	0	18	3	
Previous exposure to LFN (n, %)	139	60	338	44	27	20	505	44	< 0.0001
Reason for discontinuation									
Inefficacy	64	46	229	68	16	59	309	62	
Non-serious adverse event	69	50	104	31	11	41	184	36	
Serious adverse event	6	4	5	1	0	0	11	2	
Previous exposure to SSZ (n, %)	68	29	157	20	20	15	245	21	0.0001
Reason for discontinuation (n, %)									
Inefficacy	17	50	46	63	1	33	64	26	
Non-serious adverse event	18	47	26	36	2	66	46	19	
Serious adverse event	0	0	1	1	0	0	2	0.8	
Previous exposure to PDN ( n,%)	188	80	575	75	122	90	885	78	< 0.0001

*csDMARD* conventional synthetic disease-modifying antirheumatic drug, *DMARD* disease-modifying antirheumatic drug, *MTX* methotrexate, *LFN* leflunomide, *SSZ* sulfasalazine, *PDN* prednisone

MTX, LFN, SSZ, and GC prescription patterns at the time of initiation of the first TNF antagonist are shown on Table 3. At initiation of the first TNF antagonist, 977 patients (85 %) were receiving concomitant GCs, MTX, LFN, or SSZ: 15 % (173 patients) received their first TNF antagonist without any concomitant GC or csDMARD, i.e., true monotherapy, and 6 % received their first TNF antagonist with GCs. The drug most commonly used at the time of first TNF antagonist initiation was MTX alone or in combination (58 %), followed by PDN (45 %), LFN (24 %), SSZ (5 %), and anti-malarial agents (1 %). Over 80 % of patients were using non-steroidal anti-inflammatory drugs (NSAIDs). Mean doses of PDN, MTX, and LFN were the commonly used doses. Mean duration of the previous courses with csDMARD therapies before the first TNF antagonist were 2 years for MTX, and less than 1 year for LFN.

**Table 3:** Prescription patterns of concomitant MTX, LFN, SSZ, and PDN with the first TNF antagonist

Current treatment with synthetic DMARDs plus the first TNF antagonist	First TNF antagonist monotherapy		First TNF antagonist plus one csDMARD		First TNF antagonist plus two or more csDMARDs		Total		P*
	*n*	%	*n*	%	*n*	%	*n*	%	
	234	21	766	67	136	12	1.136	100	
None (n, %)	172	73							
PDN (n, %)	62	26	336	44	115	84	513	45	0.0001
MTX (n, %)			364	47					< 0.0001
MTX + PDN (n, %)			189	25					< 0.0001
LFN + PDN (n, %)			129	17					< 0.0001
LFN (n, %)			57	7					< 0.0001
SSZ + PDN (n, %)			9	1					< 0.0001
SSZ (n, %)			5	0.6					< 0.0001
Combinations (n, %)									< 0.0001
MTX + LFN + PDN					66	48			
MTX + SSZ + PDN					24	17			
MTX + SSZ					12	9			
LFN + CLQ + PDN					8	6			
MTX + SSZ + HCLQ + PDN					5	4			
MTX + LFN					4	3			
Others					17	13			
NSAIDs (*n*, %)	201	86	673	88	127	93	1.013	88	0.09
	Mean	SD	Mean	SD	Mean	SD	Mean	SD	
Current PDN dose, mg/day (mean ± SD)	10.7	±5.6	9.6	±6.7	10.0	±7.5	9.8	±6.6	0.002
Current MTX dose, mg/week (mean ± SD)	0	0	15.3	±4.8	16.0	4.7	15.5	4.8	0.08
Duration of current treatment with MTX (months) (mean ± SD)			31.0	32.2	21.5	23.6	27.6	28.6	0.0001
Current LFN dose, mg/day (mean ± SD)	0	0	18.8	3.1	15.6	4.9	17.8	4.0	< 0.0001
Duration of current treatment with LFN (months) (mean ± SD)			12.9	19.0	9.4	9.9	12.0	16.5	0.5
Follow up, (years) (mean ± SD)	2.1	1.0	1.8	1.0	1.6	1.1	1.8	1.1	0.0001

*csDMARD* conventional synthetic disease-modifying antirheumatic drug, *DMARD* disease-modifying antirheumatic drug, *MTX* methotrexate, *LFN* leflunomide, *SSZ* sulfasalazine, *PDN* prednisone, *NDAID* non-steroidal anti-inflammatory drug

Effectiveness data are shown in Table 4. With regard to disease activity, all treatment groups showed clinically significant improvements in final vs. baseline DAS. Physical function data assessed by the HAQ scores were significantly different between groups at the final vs. the baseline assessment, and the TNF antagonist plus two or more csDMARDs group had the poorest physical function. The percentage of patients with completed visits were 56 %, 51 %, and 60 % in each group, respectively; *p* = 0.4. The number and causes of loss of follow up were the same in the three groups except for adverse events. The adverse events were more frequent in the patients treated with the first TNF antagonist plus one csDMARD, with marginal statistical significance. Loss of follow up due to patient´s decision were due to intention to become pregnant, change of city, addition of other inter-current disease, and in some cases the reasons were unknown.

**Table 4:** Rresponse in patients with rheumatoid arthritis at 6 months and follow-up data

Treatment with synthetic DMARDs	First TNF antagonist monotherapy		First TNF antagonist plus one csDMARD		First TNF antagonist plus two or more csDMARDs		Total		Pp*
n, %	234	21	766	67	136	12	1.136	100	
Baseline DAS28 score (mean ± SD)	5.9	1.0	5.7	1.1	5.7	1.0	5.8	1.1	0.2
Final DAS28 score (mean ± SD)	3.56	1.54	3.39	1.52	3.6	1.3	3.4	1.5	0.8
Difference in DAS28 score (mean ± SD)	1.6	0.7	1.6	0.7	1.7	0.6	1.6	0.7	0.4
Baseline HAQ score (mean ± SD)	1.40	0.58	1.44	0.65	1.63	0.59	1.461	0.633	0.002
Final HAQ score (mean ± SD)	0.83	0.71	0.93	0.76	1.08	0.74	0.930	0.752	0.005
Difference in HAQ score (mean ± SD)	0.60	0.69	0.51	0.75	0.60	0.70	0.545	0.734	0.1
Follow up									
Completed last visit (n, %)	132	56	391	51	82	60	605	53	0.46
Loss of follow up (n, %)	102	44	375	49	54	40	531	47	0.07
Reasons									
Adverse event	33	32	174	46	16	30	223	42	0.001
Inefficacy	30	29	87	23	12	22	129	24	0.5
Patient decision	7	7	15	4	3	5	25	5	0.6
Unknown	32	32	99	27	23	43	154	29	0.4

*P value for between-group comparisons. Differences between groups in baseline vs. final disease activity score in 28 joints (DAS) and health assessment questionnaire (HAQ) scores were statistically significant, *p* < 0.001. *csDMARD* conventional synthetic disease-modifying antirheumatic drug, *DMARD* disease-modifying antirheumatic drug

**Fig. 2:**
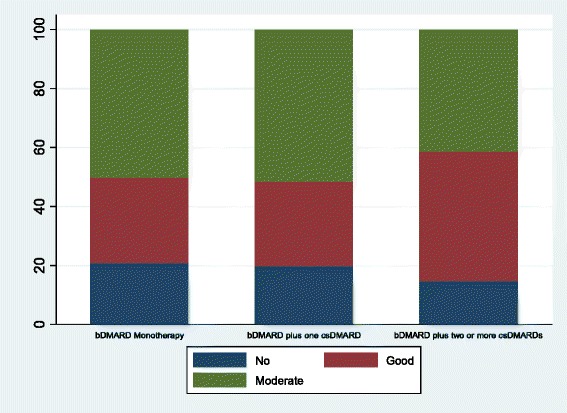
Response according to European League Against Rheumatism Criteria. c*sDMARD* conventional synthetic disease-modifying antirheumatic drug, *bDMARD* biologic disease-modifying antirheumatic drug

**Fig. 3:**
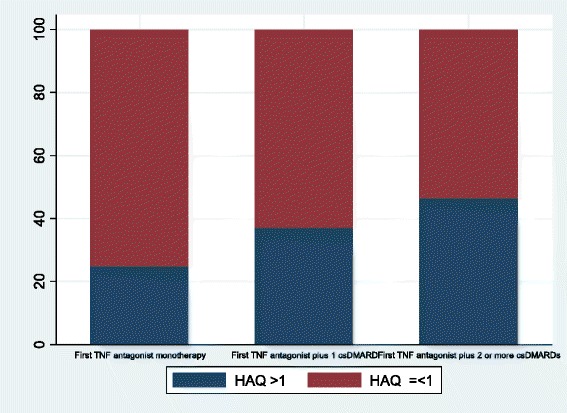
Percentages of patients with a health assessment questionnaire (*HAQ*) score > 1 and HAQ score ≤ 1. c*sDMARD* conventional synthetic disease-modifying antirheumatic drug

Percentages of patients with good EULAR response were 29 % in the first TNF antagonist monotherapy group, 29 % in the first TNF antagonist plus one csDMARD group, and 43 % in the first TNF antagonist plus two or more csDMARDs group (*p* = 0.07). Percentages of patients with moderate EULAR response were 50 %, 52 %, and 51 %, respectively, and marginal statistically significant differences were observed (*p* = 0.07) (Fig. 2). Percentages of patients with a final HAQ score ≤ 1 were 73 %, 63 %, and 54 %, respectively (*p* = 0.01) (Fig. 3).

**Fig. 4:**
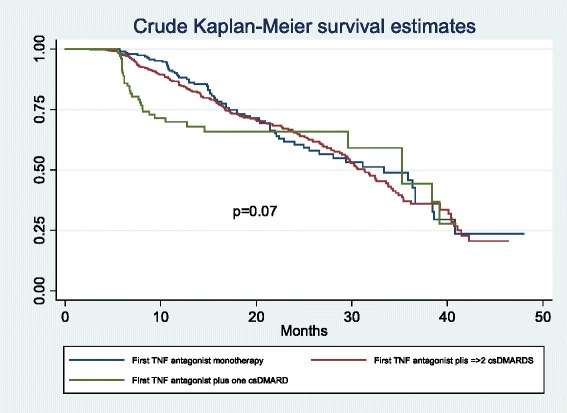
Crude survival life table. c*sDMARD* conventional synthetic disease-modifying antirheumatic drug

**Fig. 5:**
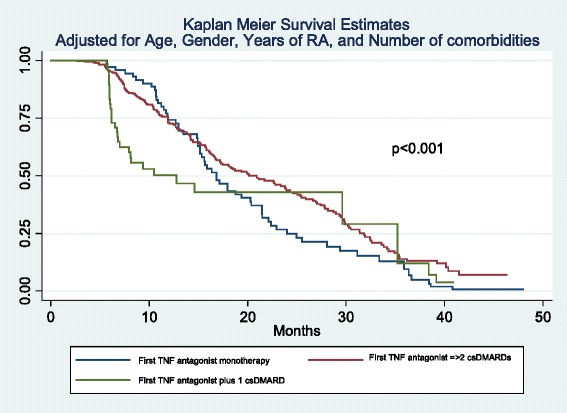
Adjusted survival life table. c*sDMARD* conventional synthetic disease-modifying antirheumatic drug

On crude analysis of drug survival, for TNF antagonist survival the mean (25^th^ to 75^th^ percentiles) was 33.3 (16.8–40.7) months in the TNF antagonist monotherapy group, 30.4 (16.6–41.4) months in the TNF antagonist plus one csDMARD group, and 34.2 (8.1–40.8) months in the TNF antagonist plus two or more csDMARDs group (*p* = 0.07) (Fig. 4). When this analysis was adjusted for age, gender, number of comorbidities, and disease duration, thus, controlling for these potential confounders, there were statistical differences in survival between the three groups, with mean TNF survival of 16.7 (11.8–24.1), 20.1 (11.3–31.3), and 10,5 (6.1–29.6) months, respectively (*p* < 0.001) (Fig. 5).

Finally, in the logistic regression the model that best explained a good EULAR response included the baseline DAS28 and the 6-month DAS28 (*p* <0.0001 and *R* ^2^ 0.8). The model was not modified after adjustment for age, gender, disease duration, and treatment group. The model followed a similar pattern using the different techniques for handle missing values (data not shown).

## Discussion

This was an observational, prospective, multicentre study of a cohort of patients with RA treated with their first TNF antagonist in standard clinical practice within the Andalusian Health Services. The registry included 90 % of prescriptions of biologic agents issued in this community. We observed differences in the characteristics of patients who received their first TNF antagonist in monotherapy or in combination with csDMARDs, and found that prescriptions were largely influenced not only by accessibility to biological drugs within the health system, but also by the clinical characteristics of the patients, particularly comorbidities and drug intolerance, and by the previous experience of the rheumatologist [14].

Patients in the three groups had differences in the type and number of comorbidities. As we know since the time of Hippocrates, there are patients, not diseases, and this is the art of medicine reflected in DMARDs prescription patterns, in spite of treatment schedules that are defined in Spain in summaries of product characteristics and their respective Clinical Practice Guidelines [1, 2], (https://www.nice.org.uk/guidance/cg79/chapter/Recommendations#pharmacological-management), [3, 19]. The data are in agreement with data recently reported by other registries and publications [5, 8–14].

In our study, before initiation of their first TNF antagonist, only 1 % of patients had not received GCs, MTX, LFN, SSZ, and/or CLQ/HCLQ. PDN, either as monotherapy or in combination with other csDMARDs was the most commonly prescribed drug (78 %), followed by MTX (50 %), and LFN (44 %); SSZ (21 %) was only used in a small number of cases. Rheumatologists prefer combinations of csDMARDs with PDN, and the most frequently prescribed for combination therapy is MTX [4–7, 12–15]. This high level of prescription of PDN at low or very low doses, in monotherapy or in combination with csDMARDs or a TNF antagonist, is a consequence of the effectiveness of this drug for reducing signs and symptoms of the disease. The advantages of PDN include improved patient-reported outcomes and lower disease activity levels, its benefit on radiological evidence of disease progression, and its very favourable efficacy/toxicity ratio producing very low rates of serious adverse events, and no serious adverse events related to this low or very low dose.

According to the literature, low or very low doses of steroids are used the same way as any other csDMARDs, and should be considered as such [8–12, 16, 17]. The frequency of baseline use of steroids found in several registries of patients with RA receiving bDMARDs are the following: 27 % in the Swiss Registry [9], 22 % in the Danish Registry [10], and in accordance with a review including various countries [11], figures range from 38 % to 84 % in Germany (84 %), Spain (52 %), Sweden (51 %), UK (44 %), and USA (38 %) (CORRONA database). Considering these data, TNF antagonists are not really given in monotherapy, due to the high frequency of GC use in patients treated with TNF antagonists in monotherapy. Unfortunately, this study did not include data on radiographic progression, so the real value of GCs as csDMARDs cannot be assessed.

The low use of SSZ in Spain may be explained by the enteric coating of the formulation. This is different from the coating used in other European countries, and its efficacy is lower than that reported in studies conducted in Northern Europe. As a consequence, SSZ is only used after other csDMARDs, namely MTX, LFN, and CLQ/HCLQ [21]. Our results may be influenced by our requirement that during the period in which the study was conducted, patients had to have failed to respond to at least two csDMARDs in order to be prescribed a bDMARD [3, 19]. A lack of efficacy, followed by mild csDMARD toxicity (30–40 %), were the primary reasons for the participating rheumatologists to request bDMARDs. Severe toxicity was very infrequent (< 3 %).

With regard to GCs and csDMARDs prescription patterns, at the time of initiation of the first TNF antagonist most patients were receiving monotherapy or combination therapy with MTX, followed by PDN, LFN, and SSZ. The decreased frequency of GC treatment, from 78 % to 45 %, needs to be emphasized. Only 15 % of patients received the first TNF antagonist without concomitant csDMARDs or GCs. Almost one third (27 %) of patients who received their first TNF antagonist in monotherapy were being treated with PDN as the sole medication. Again, the data show that low or very low doses of PDN were a commonly used treatment at the time of initiation of the first TNF antagonist. GC, MTX, and LFN doses were similar to those recorded in other registries and observational studies [5, 8–12, 14]. It should be noted that a smaller subgroup of patients (those who received their first TNF antagonist plus two or more csDMARDs) were treated with different GCs, and csDMARD combinations. The most commonly used regimen was combination treatment with PDN plus one or more csDMARDs.

It should be emphasized that patients achieved adequate responses irrespective of the treatment they received. There were no differences between groups in improvement in disease activity after starting the biologic agent, but poorer levels of physical function were observed in the baseline and final assessments of those patients treated with the first TNF antagonist plus two or more csDMARDs. After adjustment for potential confounders, the survival analysis showed the best survival rates in the group treated with the first TNF antagonist plus one csDMARD.

This study had advantages that should also be pointed out. First, the study was based on data from standard clinical practice that included 90 % of biologic drug prescriptions. This prospective study was conducted in the setting of specialized rheumatology care and was specially designed to determine prescription patterns of GCs, csDMARDs, and the first TNF antagonist. Furthermore, patients’ clinical characteristics were similar to those observed in other European registries of patients with RA.

Unfortunately, due to the large number of patients lost to follow up, effectiveness data should be viewed with reservation, even though no statistically or clinically relevant differences were found in a comparison of sociodemographic and RA disease characteristics between patients lost to follow up vs. patients who continued (data not shown). The different statistical techniques used to handle missing values also showed different results. We know that the effectiveness data are not fully robust, as they might reflect a cohort of survivors. Another limitation is that, currently, many of these patients who cannot receive csDMARDs are treated with TCZ; thus, the external validity of the data is limited to patients who cannot receive TCZ. The third handicap is the lack of evaluation of radiographic outcome.

## Conclusions

Patients receiving the first TNF antagonist monotherapy, the first TNF antagonist plus one csDMARD, and the first TNF antagonist plus two or more csDMARDs are patients with different comorbidities and disease characteristics, which influence the type of drugs they receive. Low or very low doses of GCs are frequently used. Combination treatments with PDN plus csDMARDs are more frequently used by rheumatologists, compared to csDMARD monotherapy or other combinations. In most cases, these agents are discontinued due to lack of efficacy, requiring the introduction of TNF antagonist therapy. With the first TNF antagonist, PDN in combination with one csDMARD is used in almost half of the cases. Monotherapy with a TNF antagonist without PDN or csDMARDs is uncommon. In spite of their different characteristics, patients receiving the first TNF antagonist monotherapy, the first TNF antagonist plus one csDMARD, and the first TNF antagonist plus two or more csDMARDs obtain clinical responses in terms of activity and physical function measured by an acceptable HAQ score. Survival differed in the three groups of patients.

## Abbreviations

ACPA: anti-citrullinated protein antibodies
bDMARDs: biologic disease-modifying anti-rheumatic drugs
CCT: controlled clinical trials
CLQ: chloroquine
csDMARDs: conventional synthetic disease-modifying anti-rheumatic drugs
DAS28: disease activity score with 28 count joints
DAS28-4v-ESR score: disease activity score with four variables and erythrocyte sedimentation rate
DMARD: disease-modifying anti-rheumatic drug
ESR: erythrocyte sedimentation rate
EULAR: European League Against Rheumatism
GCs: glucocorticoids
HAQ: health assessment questionnaire
HCLQ: hydroxychloroquine
LFN: leflunomide
MTX: methotrexate
PCR: protein C reactive
PDN: prednisone
PPD skin reaction: purified protein derivative skin reaction
RA: rheumatoid arthritis
RF: rheumatoid factor
SSZ: sulfasalazine
TCZ: tocilizumab
TNF: tumor necrosis factor
VAS: visual analogue scale
